# Helical Tomotherapy in Children and Adolescents: Dosimetric Comparisons, Opportunities and Issues

**DOI:** 10.3390/cancers3043972

**Published:** 2011-10-25

**Authors:** Maurizio Mascarin, Francesca Maria Giugliano, Elisa Coassin, Annalisa Drigo, Paola Chiovati, Andrea Dassie, Giovanni Franchin, Emilio Minatel, Mauro Gaetano Trovò

**Affiliations:** 1 Pediatric Radiotherapy Unit, Centro di Riferimento Oncologico- National Cancer Institute/Via Franco Gallini, 2 33081 Aviano (PN) Italy; E-Mails: francesca_giugliano@hotmail.com (F.M.G.); elisa.coassin@libero.it (E.C.); 2 Department of Radiation Therapy, Centro di Riferimento Oncologico- National Cancer Institute/Via Franco Gallini, 2 33081 Aviano (PN) Italy; E-Mails: adrigo@cro.it (A.D.); pchiovati@cro.it (P.C.); adassie@cro.it (A.D.); gfranchin@cro.it (G.F.); eminatel@cro.it (E.M.); mgtrovo.rt.cro@cro.it (M.G.T.); 3 Department of Medical Physics, Centro di Riferimento Oncologico- National Cancer Institute/Via Franco Gallini, 2 33081 Aviano (PN) Italy; 4 Seconda Università di Napoli, Napoli 80138, Italy

**Keywords:** paediatric oncology, helical tomotherapy, intensity modulated radiotherapy, 3D conventional radiotherapy, treatment planning, organs at risk, late effects, dose homogeneity

## Abstract

Helical Tomotherapy (HT) is a highly conformal image-guided radiation technique, introduced into clinical routine in 2006 at the Centro di Riferimento Oncologico Aviano (Italy). With this new technology, intensity-modulated radiotherapy (IMRT) is delivered using a helicoidal method. Here we present our dosimetric experiences using HT in 100 children, adolescents and young adults treated from May 2006 to February 2011. The median age of the patients was 13 years (range 1–24). The most common treated site was the central nervous system (50; of these, 24 were craniospinal irradiations), followed by thorax (22), head and neck (10), abdomen and pelvis (11), and limbs (7). The use of HT was calculated in accordance to the target dose conformation, the target size and shape, the dose to critical organs adjacent to the target, simultaneous treatment of multiple targets, and re-irradiation. HT has demonstrated to improve target volume dose homogeneity and the sparing of critical structures, when compared to 3D Linac-based radiotherapy (RT). In standard cases this technique represented a comparable alternative to IMRT delivered with conventional linear accelerator. In certain cases (e.g., craniospinal and pleural treatments) only HT generated adequate treatment plans with good target volume coverage. However, the gain in target conformality should be balanced with the spread of low-doses to distant areas. This remains an open issue for the potential risk of secondary malignancies (SMNs) and longer follow-up is mandatory.

## 1. Introduction

Cancer is the second commonest cause of death in children in the developed countries [1]. Incidence rates of childhood cancer have risen over the last few decades. Cure rates have also increased progressively over the last few years due to highly specific diagnostic procedures, the use of standardized chemotherapy protocols, recent studies which focused on the management of toxicities, as well as more sophisticated radiation treatments. Within the Italian population there are about 8 million children and another 6 million adolescents and young adults (AYA), so we expect to see between 260 and 350 children diagnosed with solid tumors requiring some form of radiation therapy (RT) annually in Italy. Long-term survivors in the pediatric population show an elevated risk for adverse events. The late effects in children, especially after RT, develop gradually over several months or years. They include neurocognitive deficiencies, cardiac toxicity, endocrinological problems, growth defects, and the development of secondary malignancies (SMNs). The incidence of SMNs 30 years after treatment is around 10–20%. For this reason, the use of RT is still debated in pediatric oncology.

Intensity-modulated radiotherapy (IMRT) is a new method of planning and delivering RT. In comparison to the current, well-established technique of three-dimensional conformal radiation therapy (3D-CRT), IMRT has proved to have remarkable advances in target conformity, allowing dose escalation to the target volume and sparing neighboring organs at risk (OARs). IMRT has been used with great caution in the pediatric population for several reasons. Among these, an increased fraction time, necessity for exact immobilization with tailor-made steep dose gradients and the fear of increased SMNs induction by the potentially greater low dose spillage or integral dose (ID) [2].

Helical Tomotherapy (HT) represents both an innovative RT approach and a novel treatment device that merges a linear accelerator designed for IMRT with elements of a helical computer megavoltage tomography (MV-CT) scanner. During HT treatment, a 6 MV x-ray fan beam modulated by a binary multi-leaf collimator (MLC) is delivered from a rotating gantry while the patient on the treatment couch is slowly moving through the gantry aperture, resulting in a helical beam trajectory. The MLC is equipped with 64 pneumatically driven leaves, that open and close across the slit opening. To perform the intensity modulation, at any given time, each leaf can be closed, covering a portion of the slit, or open, allowing radiation through, or changing between these states [3-5]. In addition, HT allows MV-CT imaging and image registration with the planning CT for patient alignment. Image Guided Radiotherapy (IGRT) uses daily CT scanning to create 3D images of body anatomy in order to visualize set-up errors. Other than to assure an exact reproduction of the spatial position of the patient, in some situations it allows us to monitor the tumor shrinkage or changes in the body (e.g., weight loss). Finally, HT patients can be treated in a supine position without the problem of junctions; thus resulting in more comfortable treatment, especially for children requiring sedation.

HT can potentially provide an advantage over conventional techniques in certain situations because of its ability to generate highly conformal avoidance of critical structures immediately adjacent to the tumor target. This can be shown in multiple anatomical sites.

## 2. Patient Population and Treatment Techniques

This study describes our clinical dosimetric experience with HT in pediatric and AYA patients treated at the Centro di Riferimento Oncologico Aviano, Italy. In our Department, IMRT delivered with Linac (Eclipse Varian) and HT (Hi-ART Tomotherapy) equipment were applied to the pediatric population from 2005 and 2006, respectively.

We began our experience with HT in children in May 2006 and by February 2011 we had treated 100 pediatric and adolescent patients. The median patient age was 13 years (range 1–24). The AYA patients were included because our Institute is involved in a special program for this patient population [6]. The treated sites were the central nervous system (n = 50), the head and neck (n = 10), the thoracic bone and pleural cavity (n = 9), the abdomen (n = 8), the pelvis (n = 3), limbs (n = 7), and mediastinal-neck nodes (n = 13). The most representative histology were “primary” brain tumors, followed by sarcomas (bone and soft tissue), lymphomas, neuroblastomas, nasopharynx cancers and others. Table 1 shows more detailed information about the patients' characteristics.

We chose HT when the case met at least one of the following five criteria: (1) complex tumor geometry (irregular target); (2) close proximity of OARs; (3) target volume coverage with different dose modulation; (4) noticeable tumor shrinkage during RT; (5) patients treated for an extensive planning target volume (PTV) (e.g., craniospinal irradiation (CSI), lymphoma), when the “low dose bath” was considered not much more unfavorable when compared to 3D-CRT, specifically when the ID was not completely in favor of 3D-CRT use.

### 2.1. Immobilization

Immobilization was obtained using several devices and depended on the treatment site, the patients' age, the need to minimize patient movement and setup errors, as well as to maintain the same position during treatment and assure that it could be reproduced accurately each time. Patients were positioned as comfortably as possible, as many who require RT are very young children and need sedation or anesthesia (31/100 patients). Patients with brain tumors or head and neck tumors were immobilized with individual thermoplastic masks, sometimes with an auxiliary bite block. Younger patients with thoracic or abdominal-pelvic tumors were immobilized by using a vacuum bag and customized pillows for legs and feet. The adolescents and young adults were often put directly on the treatment couch. With the introduction of HT, the patients were all aligned and immobilized in a supine position, the same for CSI [7].

### 2.2. Radiation Imaging—Contouring

**Figure 1.**
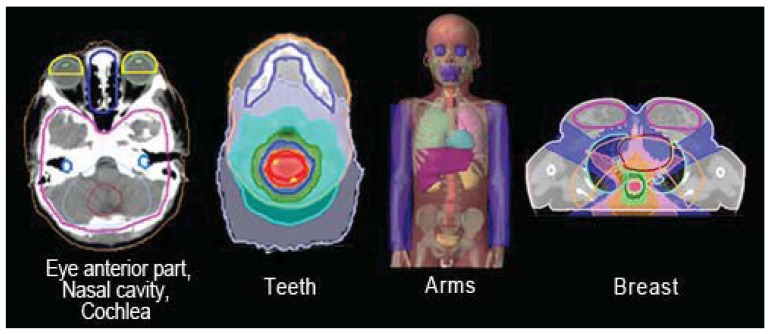
An example of contouring of different OARs marked during the HT process: anterior part of the orbits, nasal cavity, cochlea, teeth, arms, breasts.

One of the fundamental prerequisites for conformal RT and especially for all IMRT techniques is the localization of the target, starting with the gross tumor volume (GTV) and the clinical target volume (CTV), and moving outwards to the PTV. Inverse planning for IMRT-HT requires comprehensive contouring of all OARs. The CT images are acquired from a slice thickness and spacing of 5 mm. A 2.5 mm slice thickness CT is used for brain and head and neck targets. In our practice, the volume of interest is generated with a co-registered CT/MRI (magnetic resonance imaging) ± PET (positron emission tomography); starting with a multimodality diagnostic imaging set, we delineate the target and OARs, and next we proceed with treatment planning optimization. Some extra structures are generated (“tune structures”) to obtain a better optimization around the target which include e.g., the anterior part of the orbits, the nasal cavity, the jaw-maxillary-dental area, the arms, and the breasts (Figure 1). The spinal cord when considered as OAR is always contoured and automatically expanded with a 1 cm margin to create the “spinal cord tuning,” which better spares the organ.

The setup margin of PTV on CTV is not universally attributed and it depends on the tumor site, mobility of the organ involved, age and collaboration of the patient, the experience of the Center, and quality assurance procedures. Generally, we consider an expansion of 5 mm for every CTV, except for patients (fixed with mask and bite block) with head and neck lesions close to OARs (3 mm). For patients who underwent CSI, different expansions between cranial CTV (4–5 mm), and lumbar-sacral spinal canal CTV (5–7 mm) were used, depending on quality of immobilization (sedation, patient collaboration, etc.) and PTV length.

### 2.3. Treatment Planning Parameters

The HT plans were generated by the Tomotherapy planning workstation. The dose was prescribed to a PTV, assuming that 95% isodose covered all target volumes. The dose limits for critical structures were the standard values used in clinical protocol practice for pediatric tumors, using the priority, importance, and penalty factors. Parameters specified as part of the optimization/dose calculation process were pitch, beam thickness and modulation factor. The typical planning parameters were: fan beam thickness (2.5 cm; 1 cm for target close to optic regions or spinal cord), modulation factor (2.0–2.5) and pitch (0.172 or 0.215 or 0.287). A field width of 5 cm was chosen only in tall older adolescent patients requiring CSI. Briefly, HT system planning uses an interactive inverse treatment planning algorithm based on least squares minimizations of an objective function [8], and calculation grid size was selected during the optimization stage (fine, 512 × 512; normal, 256 × 256; coarse, 128 × 128). Typically the normal grid size was used. The coverage of 95% PTV volume with the prescribed dose was set as the minimum optimization objective (high penalty and high importance were set to guarantee the minimum dose to the target).

### 2.4. Pre Treatment MV-CT Acquisition

MV-CT was acquired prior to treatment in order to precisely align the patient every day. The pre-treatment MV-CT scanning was performed in 100% of HT fractions. The length of scanned area was chosen individually on the basis of anatomy of interest and target. The patient dose is about 1-2cGy for a 10 cm length scanning. Generally, we tried to avoid the scan along particularly sensitive regions like the lens. In medulloblastoma patients a double scan of about 10–15 cm was performed in the cranial-cervical region and in the lumbar region. The entire scan of CSI PTV is not feasible because it requires about 20 minutes just for the MV-CT.

The correlation of the MV-CT with the planning CT (co-registration) was done automatically with algorithms generally focusing on bone anatomy. Moreover, a manual correction was often applied to the thoracic and abdominal target. During the first three fractions the registration process was performed both by physicists and radiation therapists. From the fourth fraction onwards it was performed only by radiation therapists after they had received special training. A 3D correction vector was calculated on the basis of three setup axes: X (later-lateral), Y (cranium-caudal) and Z (anterior-posterior) axis. The Z axis was reseated after the first fraction because the flexion of the Tomotherapy couch in this direction also depends on the patient weight. The following equation was applied to calculate the vector v:

$$\text{v}=\sqrt{x^{2}+y^{2}+z^{2}}$$

## 3. Results and Discussion

The HT plans were compared with conventional 3D plans and a decision considering both PTV coverage and OARs sparing was reached. We found that HT has the potential to improve the quality of the dose distribution both in terms of dose homogeneity within the PTV (without cold-hot spots) and OARs sparing. The Dose Volume Histogram (DVH) generated with HT showed several advantages for the mean-high doses in most cases when compared with conventional techniques. In certain cases, only HT generated adequate treatment plans with good target volume coverage. However, HT is often associated with a low dose bath.

The typical RT process times were: 3–10 hours for contouring, 4–16 hours for planning, 6–22 minutes beam-on radiation time, 15–45 minutes room time. HT requires more time in the development of different RT steps: time of simulation, target and OARs delineation, planning, delivery and verification.

For 42 patients treated for brain or head and neck tumors (1020 HT fractions) the mean detected setup correction vector was analyzed. It was 2.76 mm, 3.18 mm and 4.34 mm for mask and sedation, mask and bite block, and mask alone, respectively. In the CSI we found a different Z value between the cranial and the lumbar tract setup, probably due to the different couch flexion. The 3D correction vector for the lumbar tract, analyzed in 16 CSI patients, was 5.9 mm. However, the corrections to the lumbar tract should be done carefully because any translational movement in this region could have a negative impact on the eye area, putting it in a high dose region. To avoid this, rather than correct the cranial and lumbar tract with different setup parameters, we decided to apply a different PTV margin expansion between cranial CTV (4–5 mm) and lumbar-sacral spinal CTV (5–7 mm). So the setup errors in the lumbar spinal region were preferentially corrected only along the X axis, manually adjusting the jaw and keeping the head still. In the following sections, we describe our HT experience in various anatomical sites individually.

### 3.1. Brain Tumors

**Figure 2.**
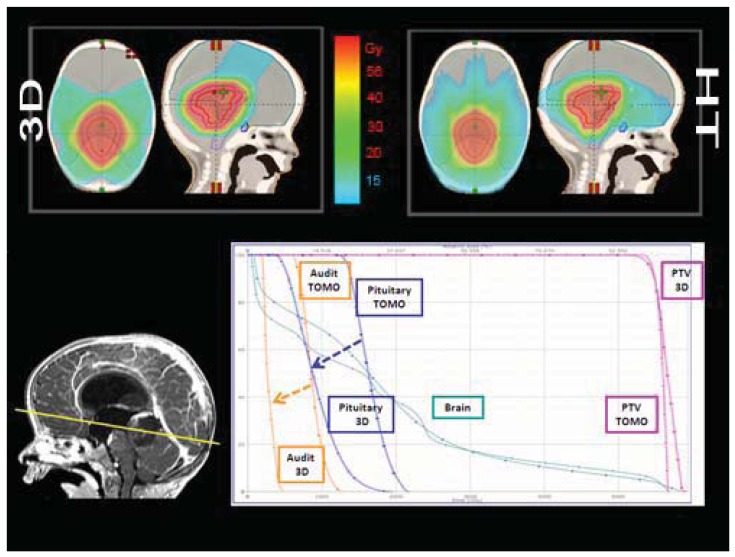
A comparison between no-coplanar 3D-CRT and Tomotherapy plans is shown in a 2-year-old male affected by atypical teratoid rhabdoid brain tumor. The DVH analysis for pituitary gland and audit and total brain are in favor of 3D-CRT.

**Figure 3.**
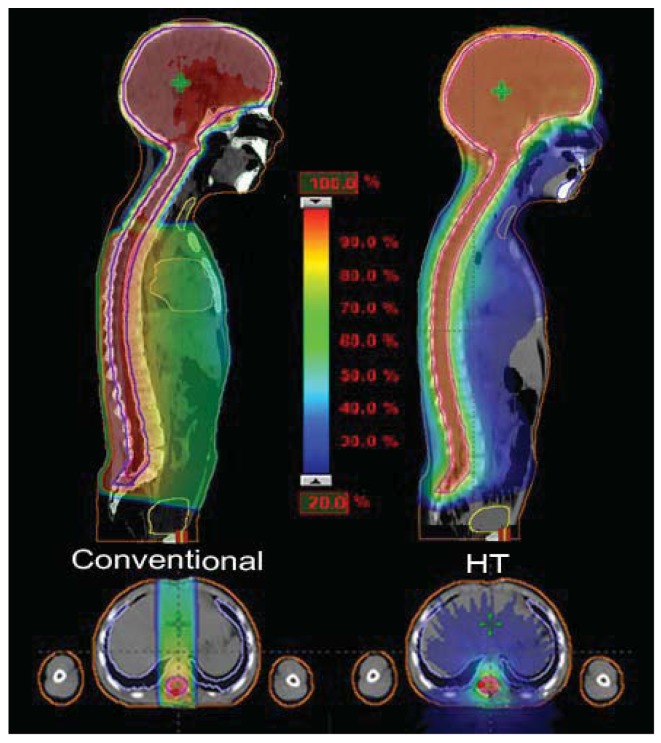
In a 4-year-old medulloblastoma patient, CSI delivered with HT, compared to conventional techniques, is able to give a more homogeneous dose and better conform the dose to the target, but with a larger low dose bath.

Structures contoured as OARs for the brain patient group were both parotids, teeth, the mandible (including temporo-mandibular joint), the spinal cord, optical structures (optical nerves, chiasm, eyes, lens), the brain, the brain stem, the pituitary gland, temporal lobes, the cochlea, and the thyroid gland. The choice of sparing one organ‘more than another’ is a complex clinical and technical challenge. The use of delivery systems with a very high degree of freedom, such as HT, could permit us to explore the potential of sparing other structures and tissues that normally cannot be efficiently spared with more conventional 3D-CRT or IMRT techniques [9]. Examples of OARs are the cochlea and the pituitary gland in the treatment of the brain. Much importance is given to the prevention of hearing loss as it could compromise the quality of life of these young and very young patients, especially in the workplace and during social relations. Despite its small size (mean volume 0.14 cc), the cochlea is easily identified on CT planning with 3 mm cut [10]. We slightly expanded the anatomic cochlea contour as an OAR to facilitate its preservation from excessive radiation because of its small size. In fact, the value of data resulting from HT planning optimization is not so accurate for OARs whose volumes are lower than 2 cc. Neuroendocrine disturbances in anterior pituitary hormone secretion are common following radiation damage, the severity and frequency of which correlate with the total radiation dose delivered to the hypothalamus-pituitary axis and the time that has elapsed since treatment. Classically, growth hormone (GH) is the most sensitive of the anterior pituitary hormones to irradiation, followed by gonadotrophins, adrenocorticotrophic hormone (ACTH) and thyroid-stimulating hormone (TSH). The somatotrophic axis is the most vulnerable to radiation damage and GH deficiency remains the most frequently seen endocrinopathy. In our example of a 2-year-old male with an atypical teratoid rhabdoid tumor of the quadrigeminal region, the HT plan has been compared with 3D-CRT using no-coplanar fields. The tightly conformal dosimetric characteristics of HT were not advantageous with respect to the cochlea and pituitary gland DVHs. In this case, the relatively small volume of treatment, the regular target volume and the opportunity to choose the entrance fields with CRT, favored applying the latter option (Figure 2). Some patients with brain tumors need CSI and, in this case, HT has a frequent application. In our experience in a 4 year-old-male affected by medulloblastoma treated with HT (Figure 3), an inspection of DVH reveals excellent conformal quality both for CTV brain and spinal cord with better sparing of OARs close to the target [7]. In comparison with conventional techniques, CSI delivered with HT is able to achieve better dose homogeneity and conformality in the target volume. With HT-CSI lower doses are distributed to larger volumes and higher doses to smaller volumes, with higher doses confined to a very small volume. The potential drawback of the low dose bath is that it could have an effect on acute toxicities (e.g., on the lung, on the gastrointestinal tract) and on the total body ID [7].

The pulmonary toxicity has been studied by Penagaricano *et al.* They found no acute pulmonary toxicities in 18 patients (age 2.5–21 years) treated with HT-CSI; 11 of them had ≥50% of the lung volume that received ≥10Gy. The same author reported no high grade acute toxicity profiles: weight loss (14/18 patients, grade 1–2) and nausea (10/18, grade 1–2) were the most common acute toxicities [11].

On the contrary, the total body ID slightly increases in comparison to conventional techniques delivered with linear accelerator. Based on our experience, in 15 children younger than 8 years treated with CSI for different brain tumors, the total body ID showed a difference of about 11% in favor of 3D-CRT-CSI when compared to HT-CSI [12,13]. However, results for ID in CSI vary in the literature. Shi *et al.*, in a single patient study, showed that the HT plan produces lower non tumor ID when compared to the step-and-shoot IMRT plan, and better homogeneity for the spinal PTV [14]. In a comparison between HT and conventional CSI, Penagaricano *et al.* found an ID 8% higher in two patients, but 2% lower in another one [15]. Finally, Sharma *et al.*, in a dosimetric study in 4 pediatric and adolescent patients, reported that HT-CSI was able to reduce the ID in 4 of the 10 analyzed OARs (heart, thyroid, liver and esophagus) when compared to 3D-CRT-CSI. The authors focused their study on OARs, but the total body ID was not reported [16].

**Figure 4.**
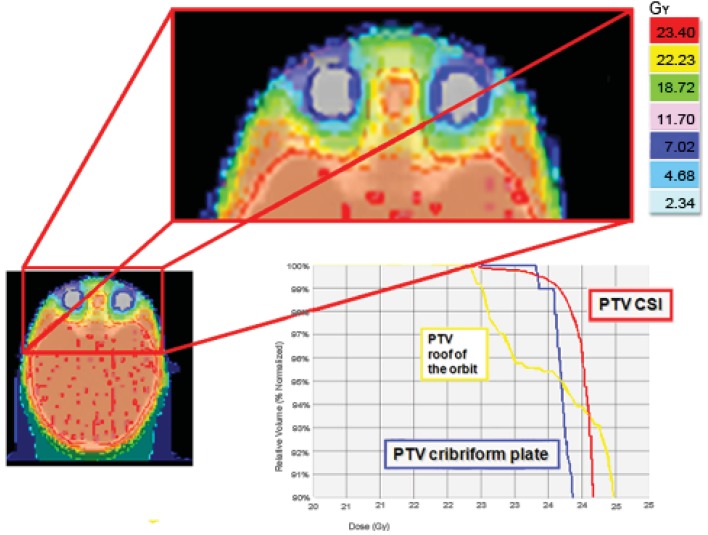
An inspection of a central axis slice through the eye level shows that the cribriform plate is not covered as well as the rest of the craniospinal PTV. For this reason we usually build some extra PTV to better control these critical areas (cribriform plate PTV).

The cribriform plate is a possible site of meningeal relapse. Adequate coverage of this structure means that superior orbital tissue is included in the treatment field. An inspection of a central axis slice through the eye level shows a good ocular sparing with HT, with a 25 mm fan beam and by building a “tuning eye structure” in the anterior-bulbar space. However, the cribriform plate is not covered as well as the rest of the craniospinal PTV. For this reason we usually build some extra PTV to better control these critical areas. A good result both in the ocular area and the cribriform plate PTV was also obtained with a 10 mm fan beam, but with a total treatment time of about 40 minutes; unacceptable for a child treated daily with sedation (Figure 4).

Hypothyroidism is another common late effect, not only after CSI, but also in neck/mediastinum irradiation. The frequency of compensated hypothyroidism is reportedly as high as 43.8% among adults and 80% among children after neck irradiation. Thyroid dysfunction may develop from a few months to several years after patients have completed their RT. In children with chronic diseases, or given lengthy anti-neoplastic treatments, recurrent or persistent endocrine disorders may have a negative effect on the growth and the development of a child into adulthood [17]. With regard to the thyroid in conventional 3D CSI, the upper part of the gland received, with two cranial opposed fields, about 20% of the delivered dose and the lower part, with direct posterior field, between 50% and 70% of the delivered dose. In the HT plans, 90% of the thyroid volume received lower than 23% of the delivered dose (Figure 3).

HT-CSI provides a dosimetric advantage in the exit dose in the pelvic-bladder area when compared to conventional techniques (<5% and ≈10% of dose delivery with HT and with Linac-based conventional CSI, respectively). This is due to the divergent posterior spinal field used with the Linac, being liable for a higher dose in the anterior part of the pelvis. Differently, with HT the helicoidal fields are substantially orthogonal to the spine, and the gonadic region could be the object of OAR planning optimization. This result may be of interest to better spare the ovaries in a female patient treated with CSI, even if the gonads could be difficult to contour.

Finally, HT gives us the opportunity to re-treat areas that have been already treated. The advantages for re-treatment with HT are the greater conformality of dose distribution and the possibility to respect dose constraints for adjacent, critically sensitive, previously irradiated normal tissues. This opportunity could be of interest both for palliative intent and for patients in which curative treatment could not be obtained with other procedures. We use HT for the re-treatment of local relapsed brain tumors and “in field” relapsed Hodgkin lymphomas. Both these situations can adequately be managed by other techniques like Linac-delivered IMRT or stereotactic treatment. An unusual condition in which HT can play a specific role is the re-irradiation of the craniospinal axis. We employed this technique in a 10-year-old male with diffuse meningeal spread of disease, 24 months after the first-line CSI for a standard risk medulloblastoma, proved to be refractory to salvage chemotherapy. He received 23.4Gy in 13 fractions to the craniospinal axis, with a reduced dose to posterior fossa of 18Gy. HT allowed us to adequately re-treat the entire axis, while giving a safe dose to the posterior fossa, previously treated by the full dose (55.8Gy).

### 3.2. Head and Neck Tumors

The conventional treatment technique for head and neck tumors is composed of two phases. Phase I consists of two lateral opposed fields for the primary tumor and enlarged neck nodes, together with a lower anterior field for the lower cervical nodes. Phase II is used after 40 Gy to shield the spinal cord; usually in this phase the posterior neck nodes are treated with electron fields.

**Figure 5.**
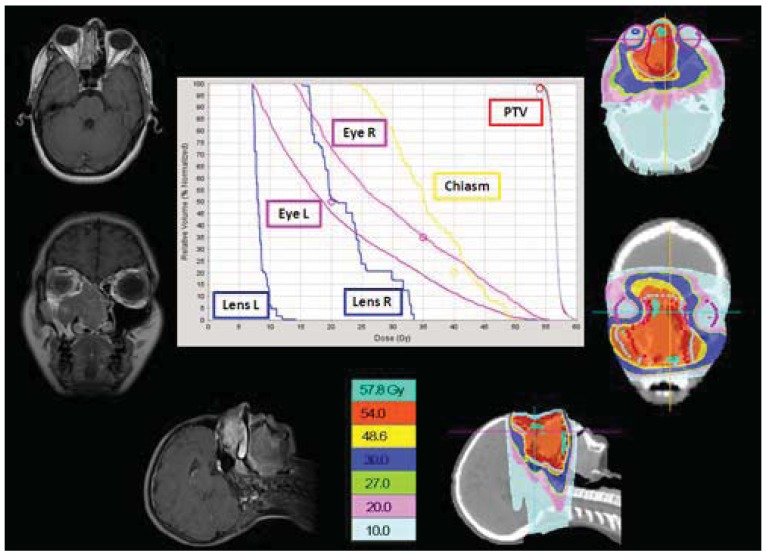
HT plan in an adolescent female affected by esthesioneuroblastoma of the nasal cavity offered good sparing of the contralateral optic nerve, chiasm and lens.

In our patients with head and neck tumors the use of HT, as an alternative to 3D-CRT, was chosen to avoid multiple fields, different energies, and junctions and to spare unavoidably higher dose to the optic nerves, chiasm, eyes and lens, as shown in our adolescent patient affected by esthesioneuroblastoma of the nasal cavity (Figure 5). HT gives us the opportunity to “paint” the high-dose region around the target volume and thus spare at least part of the mucosa from the high-dose region.

Cases like this demonstrate that HT-delivered IMRT may provide superior dose homogeneity and dose conformality when compared to earlier technologies, such as 3D-CRT or conventional RT, leading to efficient sparing of the spinal cord, the parotids, the teeth and the mandible.

In addition, we are investigating the sparing of pharyngeal mucosal structures and other tissues and organs, such as larynx, thyroid, inner ear and cerebellum. This is done to reduce the potentially dose-limiting toxicities. We pay special attention to mucosal-sparing techniques to prevent malnutrition and treatment breaks. Indeed, some authors suggested that in pediatric nasopharyngeal carcinoma the use of IMRT resulted in a significant reduction in the incidence of high grade toxicity, delayed the onset of moderate toxicity, resulting in a reduction in the total time required to deliver RT compared to CRT [18].

**Figure 6.**
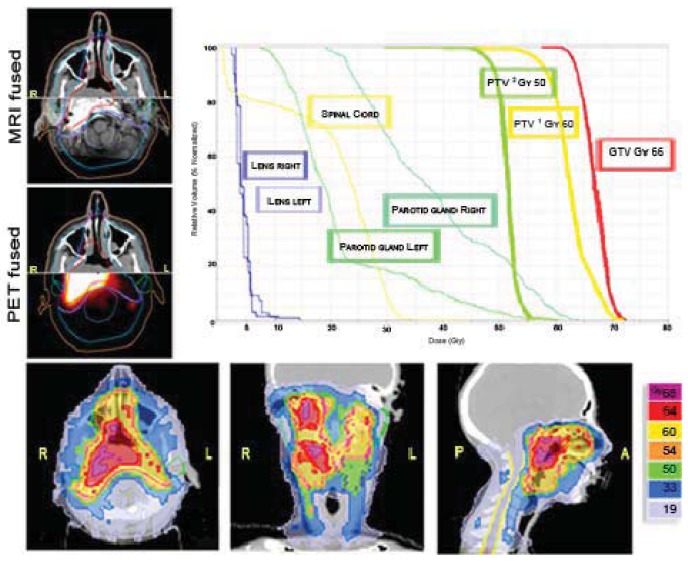
An example of nasopharyngeal cancer in a 14-year-old female in which a simultaneous integrated boost (SIB) was delivered. 66Gy/30 fractions to the GTV, 60Gy/30 fractions to the PTV1 (high risk CTV + 5 mm margin), 50Gy/30 fractions to the PTV2 (low risk CTV + 5 mm margin).

An important point in favor of HT (and other IMRT methods) is the possibility to efficiently and easily deliver different doses at different volumes (Figure 6). The choice between IMRT delivered with Linac or with HT is random for head and neck tumors in our Department. Based on our adult experience, there is no difference between the two IMRT modalities in terms of loco-regional control and development of severe, acute, and late toxicities [19]. For both techniques the patient setup is done with CT images (MV-CT or cone-beam-CT).

### 3.3. Thoracic Tumors

**Figure 7.**
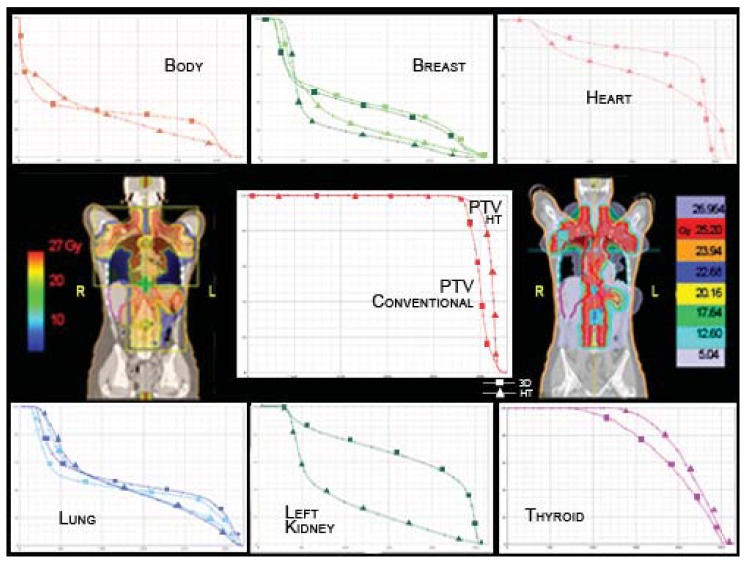
20-year-old female, Stage IIIA HL, treated with 25.2Gy/14 fractions at the end of chemotherapy. HT has dosimetric advantages compared to the conventional technique in several OARs, whereas whole body HT results in a disadvantage at lower doses and an advantage at higher doses. The total body ID is 9% lower for HT. The HT-DVHs for OARs are marked with triangles for HT and with squares for conventional RT.

RT for advanced Hodgkin Lymphoma (HL) often requires large fields and may result in significant exposure of normal tissues to ionizing radiation. Advances in the treatment of HL have resulted in a large number of long-term survivors at risk for the serious late effects of therapy. Currently, second cancers are also the primary cause of mortality among these patients with breast cancer being the most common solid tumor among women. The largest excesses of breast cancer are observed among women diagnosed with HL at age 30 years or younger, a pattern that is consistent with the known radio-sensitivity of the breast at young ages [20]. The incidence of breast cancer has been reported to increase by a factor of 4.3 (95% CI: 2.0–8.4) for patients treated with mantle irradiation [21]. While the dose response for radiation above 10Gy remains uncertain, carcinogenesis after radiation is exacerbated by the large dose gradient across the breast and treatment field position. Although HT might significantly decrease high doses delivered to the breast, it increases the volume that receives lower doses, which has also been implicated in the carcinogenesis process [22]. Conventional 3D-CRT delivered with opposite anterior-posterior fields has been successfully used to treat this disease but treatment delivery often requires photon-photon or photon-electron matching, utilization of field-in-field techniques and partial transmission blocks [23]. Dose reduction in the thyroid, breast, but also in the heart, kidney and bowel should be helpful. Based on our experience, HT obtains a greater dose homogeneity in the PTV and has dosimetric advantages compared to the conventional technique in several OARs. The most striking results have been obtained for the left breast (10.82Gy and 7.9Gy mean dose for 3D-CRT and HT, respectively), the right breast (10.13Gy and 8.73Gy mean dose for 3D-CRT and HT, respectively), the heart (19.89Gy and 17.1Gy mean dose for 3D-CRT and HT, respectively), and the left kidney (17.9Gy and 8.9Gy mean dose for 3D-CRT and HT, respectively) (Figure 7). To achieve these results we did not perform a full blocking of the structures. We applied a high importance with a very low dose constraint to the specific OAR. In our cases, this approach allowed us to achieve analogous results to full blocking, but with better optimization of the target. Instead, we use a directional block e.g., in total pleural irradiation to spare the contralateral lung and in CSI to avoid irradiation through the arms.

**Figure 8.**
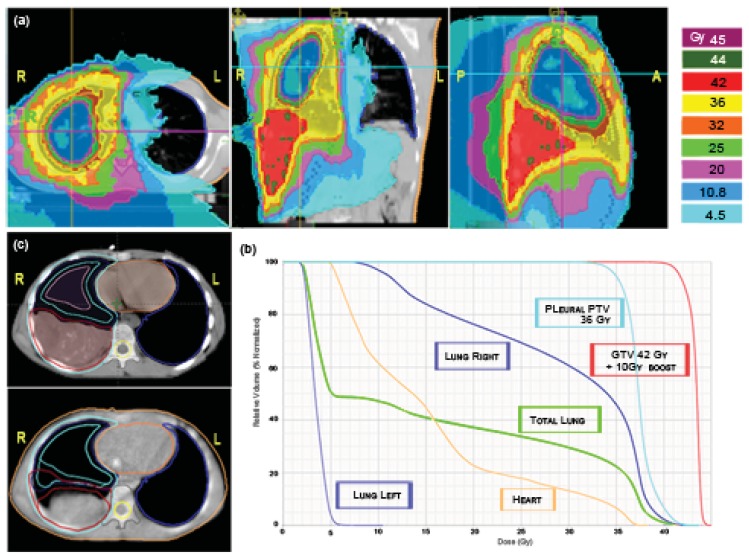
A 10-year-old male affected by right pleural PNET. HT allows different dose gradients between the pleural cavity (36 Gy/20 fractions) (**a**) and residual disease (42 Gy/20fractions) (**b**). DVHs of the first phase of treatment for the residual GTV, right pleural PTV, right lung, left lung, total lung volume and heart are shown (**c**). Further 10 Gy/5 fractions were delivered only to the shrinking tumor (bottom left) after the first phase of treatment on the basis of MV-CT acquisition.

Irradiation of the pleural cavity represents a special challenge for radiotherapists because every conventional technique determines the risk of delivering high doses to the involved lung. Even if this treatment is mostly applied in the adult population with mesothelioma, sometimes also pediatric age cases of soft tissue tumors can involve the entire pleura. We applied HT to an adolescent patient affected by PNET (primitive neuroectodermal tumor) of the right pleural cavity with multiple nodular localizations and, after chemotherapy, a residual bulky disease along the base of the diaphragm. The patient was simulated in a supine position with arms overhead and fixed with a vacuum bag. The prescription to the right pleural PTV was: first phase, 36Gy in 20 fractions with a simultaneous integrated boost of 42 Gy in 20 fractions to the post-chemotherapy residual disease [Figure 8(a)]; second phase, 10 Gy in five fractions (total dose 52 Gy) delivered only to the shrinking residual costal-diaphragmatic tumor [Figure 8(c)]. The planning was built with the following constraints: mean total lung dose <20 Gy; V20 Gy total lung <30–35%; left healthy lung, all volume <15 Gy, V5 Gy <50%; heart V 20Gy <50%. A tune structure was built in the central part of the affected lung with a dose constraint of 20 Gy [24]. The result was quite good both in terms of PTV coverage and the sparing of the contra-lateral lung and other OARs [Figure 8(a)]. The mean total lung dose and the V5 Gy were 15.9 Gy and 50%, respectively. The mean dose for the affected lung was 29.1 Gy. The maximum dose, the mean dose and the V5 Gy for the healthy lung were 10.5 Gy, 3.4 Gy and 3.8%, respectively. The maximum dose, the mean dose and the V20 Gy for the heart were 38.2 Gy, 15.5 Gy and 22%, respectively [Figure 8(b)]. The plan was initially defined on the basis of pre-RT imaging, but this could not accurately reflect the degree of normal lung exposure during all treatment. For this reason, while monitoring tumor shrinkage with daily MV-CT, we planned the second treatment phase on the basis of MV-CT acquisition, applying an adaptive therapy to try to further reduce any exposure to the normal lung [Figure 8(c)]. The patient is in complete remission after 42 months from end of RT. He developed a transient radiation pneumonitis in the right lung during the first year, requiring steroid support.

### 3.4. Abdominal and Pelvic Tumors

There are several obstacles to treating young patients with whole abdominal and pelvic irradiation. The conventional technique is not only associated with high incidence of toxicity, but also with poor target volume coverage and significant dose heterogeneity because of shielded kidneys and liver as dose limiting organs. For this patient group, contoured organs were the kidneys, the spinal cord, the liver, the spleen, the rectum, and the bladder. HT is feasible and fast for whole abdominal irradiation; this technique provides excellent coverage of the PTV and effective sparing of the OARs. The goal in advanced abdominal disease is to treat the retroperitoneal lymph nodes and the peritoneal surface while reducing the dose to the kidneys and the bone marrow. Typically, 15 Gy in 10 daily fractions are given to the whole abdomen for patients with Wilms' tumor with post surgical unresectable peritoneal implants or tumor rupture. After the first 12 Gy, the residual healthy kidney is shielded with a block. This technique results in an under-dosed abdominal area in front of the healthy kidney. Instead with HT, the abdominal cavity is treated uniformly well with a dose to the healthy kidney less than 40% of the prescribed dose. HT provided adequate coverage of the peritoneal cavity while limiting the dose to the residual kidney, spinal cord and bone marrow.

In our Center, a low radiation dose is used to treat the flank in neuroblastoma (21 Gy/14 fractions) and Wilms' tumor (14.4 Gy/8 fractions). This dose range could be responsible, if delivered to a very young child, for abnormalities in bone growth; especially in vertebral bone with scoliosis as a consequence. Generally, conventional treatment in abdominal neuroblastoma and Wilms' tumors includes the tumor bed plus 2 cm margins, the entire width of the vertebral body and para-aortic nodes in the antero-posterior fields. However, a series from Iowa University showed, in 55 children receiving megavoltage RT as part of treatment for Wilms' tumor, an incidence of scoliosis after 10 to 12 Gy, 12.1 to 23.9 Gy, and 24 to 40 Gy at 8%, 46% and 63%, respectively [25].

**Figure 9.**
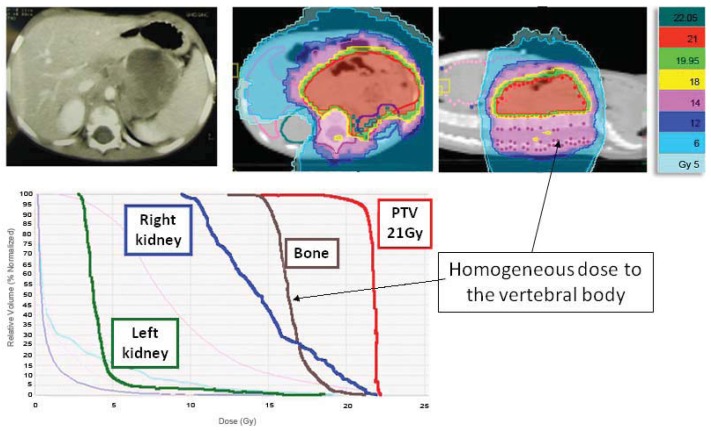
A 3-year-old male with left adrenal gland neuroblastoma treated with HT. Other than a good PTV coverage, the patient was given a homogeneous dose to the vertebral body to reduce the risk of asymmetrical growth.

**Figure 10.**
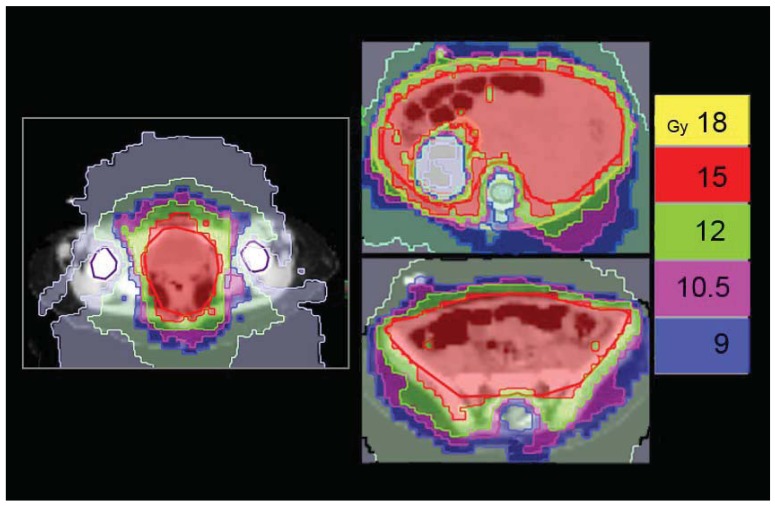
A 2-year-old female with left kidney Wilms' tumor and preoperative rupture in the abdomen. HT allows a greater homogeneity in whole abdomen irradiation with concomitant sparing of the healthy kidney to less than 40% of the prescribed dose. The technique provided adequate coverage of the peritoneal cavity while limiting the dose to the residual kidney, spinal cord and bone marrow.

Beyond a better control of the PTV dose coverage, HT enables us to further reduce small bowel dose to avoid any serious acute lower gastro-intestinal toxicity, while achieving a very homogenous dose along the vertebral body (Figure 9). In addition, there is a greater homogeneity in whole abdomen irradiation with concomitant sparing of the healthy kidney (Figure 10), which is also shown in other studies [26,27].

## 4. Conclusions

HT plays a very important part in the history of IMRT and could become a good option for children and young adult patients. In our study, we have proposed some examples of treatment with HT and our preliminary experience suggests a greater sparing of critical normal structures and a better PTV homogeneity using HT-based IMRT when compared with 3D-CRT. The dose conformity advantages of HT are sufficient to selectively recommend its use in the pediatric population. We could choose HT when the target/tumor is critical and where the margin of safety (from GTV/CTV to PTV) around the tumor is narrow, when OARs are so near the target they are at higher risk for radiation damage. Moreover, the potential for dose escalation may translate to a better local control without increasing complication rates. The use of daily image guidance requires more time than conventional RT, but it has a major impact on the verification and setup correction. This is true in all patients but especially in the younger ones, in whom treatment compliance is not always adequate. On the contrary, the increase of low doses to normal tissues and the ID demand attention and need to be evaluated with further research.

In recent decades, survivors of pediatric cancers have experienced a high incidence of chronic health problems, including secondary cancers, cardiac toxicity, fertility problems, and so on. Even if the adoption of high conformal RT techniques for the pediatric population has allowed reduction of side effects due to high doses to OARs (alopecia, bone deformities, defect of muscular growth, brain damage, etc.), caution is still required. When we adopt a new technology careful considerations are necessary, particularly in a field where the incidence of late second cancers is becoming a dominant concern. Long-term survivors of childhood cancer who received RT are at a significantly increased risk for the development of SMNs: the incidence of SMNs is around 10%-20%, 30 years after treatment [28]. Some authors estimate that the risk of induced cancer could be doubled by new techniques, such as IMRT [29]. When we analyze the risk associated with RT, it demonstrates different dose-response curves for specific secondary cancers. For example, the risk of thyroid cancer increases with low doses and, subsequently, it decreases with higher doses [30]. The low-dose data for radiation-induced carcinogenesis are taken from atomic bomb survivors and the high-dose data are gathered from RT patients. However, that outcome is not in accordance with clinical experience, in which the majority of second induced tumors occurs in or close to the high-dose treatment volume [31]. Although proton therapy may be the better treatment approach in terms of secondary cancer reduction and normal tissue sparing, its associated stray radiation (protons and neutrons outside the target) could still be strongly associated with SMNs. So dose, by itself, is a poor biomarker for SMNs risk. However, the issue regarding the increase of low-dose radiation exposure observed with HT and the supposed risk of radiation-induced cancer, needs to be further addressed. Our aim is to continue to compare HT and 3D-CRT and to establish more detailed selection guidelines for the utility of HT in the pediatric population.

## Figures and Tables

**Table 1.** Patients' characteristics: age at the time of RT, histologies, site of primary tumor, re-treatments.

**Median age (range)**	**13 (1–24 years)**
Diagnosis (n)	
Tumors of the central nervous system:	50
Medulloblastoma/PNET	23
Ependymoma	7
Low grade glioma	7
High grade glioma	6
Germ cells tumor	4
Atypical teratoid rhabdoid brain tumor	3
Sarcomas:	22
PNET/Ewing's sarcoma family	9
Rhabdomyosarcoma	7
Synovial sarcoma	4
Osteosarcoma	1
Chordoma	1
Lymphoma	13
Neuroblastoma	6
Undifferentiated nasopharyngeal carcinoma	4
Wilms' tumor	2
Others	3

Location (n)	
Central nervous system (Craniospinal irradiation)	50 (24)
Mediastinal-neck nodes	13
Head and neck	10
Thoracic bone and pleural cavity	9
Abdomen	8
Limbs	7
Pelvis	3

Re-treatment (n)	4
